# COVID-19 Prevention and Treatment—2nd Edition

**DOI:** 10.3390/life14091126

**Published:** 2024-09-06

**Authors:** Silvia De Francia, Daniela Di Grazia, Maura Caudana, Sarah Allegra

**Affiliations:** Department of Clinical and Biological Sciences, University of Turin, Regione Gonzole 10, 10043 Orbassano, Italy; daniela.digrazia@unito.it (D.D.G.); mauracaudana98@gmail.com (M.C.); sarah.allegra@unito.it (S.A.)

We have learned that the story of COVID-19 is not so simple. COVID-19 is not a pandemic; it is a syndemic. This indicates that a wider approach is needed to protect the health of our communities from COVID-19 infection. This Special Issue arises from the need for a new approach to tackle the virus both from a clinical/therapeutic and social point of view. This second edition of the Special Issue “COVID-19 prevention and treatment” published in *Life* (ISSN 2075-1729), belonging to the section “Epidemiology”, collects a series of original research and review articles related to various aspects of COVID-19. This Special Issue closed on 31 December 2023: this issue contains 14 articles, and it has been viewed 25,272 times (accessed on 29 August 2024). 

The following is an overview divided into four different themes treated in this collection of papers.

## 1. Treatment

Nine^1–9^ of the papers collected in this Special Issue focus on one of the most important topics related to the COVID-19 pandemic: how to treat it. Four^1–4^ out of these nine are focused on drug treatment, two^5,6^ are related to convalescent plasma use, one^7^ paper focuses on extracorporeal membrane oxygenation (ECMO), one on endotracheal intubation (ETI)^8^, and the last one^9^ provides the ability to interpret the global effectiveness of COVID-19 treatment. Two^5,6^ out of these nine papers are reviews. The drugs listed in papers by Cocuz et al., Sambugaro et al., Allegra et al., and Burger et al.,^1–4^ are inhibitors of viral RNA polymerase (Remdesivir), biological drugs targeting type 2 inflammation (Omalizumab, Mepolizumab, Benralizumab, and Dupilumab), glycopeptide antibiotics (Teicoplanine), and a humanized monoclonal antibody directed against interleukin-6 receptors (Tocilizumab). The studies presented are related to experiences both in children and in adults, focused on the management of respiratory and allergic disorders caused by COVID-19 infection, analyzing the potential increased risks of comorbidities, and revealing the sex and gender aspects involved in drug treatment. Two^5,6^ out of these nine papers on treatment are related to the use of convalescent plasma, which was also a topic of interest in the first edition of this Special Issue “COVID-19 Prevention and Treatment”, where three papers discussed the use of convalescent plasma. The two papers on convalescent plasma published in this Special Issue are two reviews from the same authors (Franchini and Focosi), investigating the narrative role of this measure in COVID-19 treatment. The other two papers^7,8^ on COVID-19 treatment are related to the use of different techniques, such as ECMO and ETI, reporting the increased need for these techniques for the good management of COVID-19 infection (ECMO) and, especially, the need for a better use of these techniques (ETI), to avoid laryngotracheal complication with can impact breathing, phonation, and swallowing in patients. The last paper relates to COVID-19 treatment^9^, as already mentioned, and interprets the global effectiveness of COVID-19 treatment, reporting that methodological biases are common in observational studies evaluating treatment effectiveness, and, for this reason, the development of tools useful to improve traditional statistical methods is important.

## 2. Prevention

Two out of fourteen papers focus on COVID-19 prevention, arguing considerations on vaccination efficacy^10,11^. The first paper explores the usefulness of the vaccine showing the protective role of vaccination in myasthenic patients, even if anti-CD20 therapy might be associated with a poor immune response to vaccines. The second one, through a machine learning classification, explores the identification of antibodies associated with decreased clinical immunity based on populations with different time spans after vaccination. These antibodies have important implications for maintaining long-term immunity to the severe acute respiratory syndrome coronavirus 2 (SARS-CoV-2). The paper by Qing-Lan et al. uncovers the key changes in antigen-reactive antibodies over time after vaccination, showing the potential to improve vaccine efficiency.

## 3. Mechanism of Action

One^12^ out of fourteen papers in this Special Issue focus on the mechanisms of action for COVID-19 prevention. This paper is the second in the collection reporting the use of machine learning as a research methodology. This study’s aim is to identify the important biomarkers at the expression level associated with the SARS-CoV-2 infection-mediated loss of taste or olfactory ability. In the paper by Ren et al., a machine-learning-based approach is designed to analyze the transcriptome of 577 COVID-19 patient samples, including 84 COVID-19 samples with a decreased ability to taste or smell and 493 COVID-19 samples without impairment. This study provides a new computation analysis, suggesting the latent biomarkers for predicting olfactory and gustatory impairment caused by COVID-19 complications.

## 4. General and Specific Conditions of Pathology 

Two last papers considered in this Special Issue focus on the general and specific conditions of COVID-19 pathology^13,14^: The first one is a review focused on ageusia. It is a bibliometric study analyzing the entire ageusia research literature indexed, which has also increased recently, assessing the narrative role of this symptom in the outcomes and history of COVID-19. The last paper, by Warner et al., focuses on swallowing. This paper deepens the importance of using best practices for swallowing assessments in COVID-19 patients. COVID-19 has altered clinical practice patterns worldwide, leading to the avoidance of infection and changes in some practices. COVID-19 patients are a vulnerable population that needs even more attention: the return to some instrumental assessment, as demonstrated by Warner et al., is useful.

In conclusion, the second edition of this Special Issue “COVID-19 prevention and treatment” collects papers focused on different, often lateral, aspects related to COVID-19, reinforcing the need to adapt the treatment of patients as widely as possible. 

Our thanks go to all contributors for having enriched this collection with original information and well-structured observations.

